# Ticks and Tick-Borne Pathogens in Recreational Greenspaces in North Central Florida, USA

**DOI:** 10.3390/microorganisms11030756

**Published:** 2023-03-15

**Authors:** Chanakya R. Bhosale, Kristen N. Wilson, Kimberly J. Ledger, Zoe S. White, Rayann Dorleans, Carrie E. De Jesus, Samantha M. Wisely

**Affiliations:** 1 Department of Wildlife Ecology and Conservation, University of Florida, Gainesville, FL 32611, USA; 2 Dr. Kiran C. Patel College of Osteopathic Medicine, Nova Southeastern University, Fort Lauderdale, FL 33328, USA; 3 Emerging Pathogens Institute, University of Florida, Gainesville, FL 32610, USA

**Keywords:** ticks, tick-borne pathogens, tick ecology, public health, veterinary health, Florida, Alachua County

## Abstract

Tick-borne infections are an increasing medical and veterinary concern in the southeastern United States, but there is limited understanding of how recreational greenspaces influence the hazard of pathogen transmission. This study aimed to estimate the potential human and companion animal encounter risk with different questing tick species, and the bacterial or protozoal agents they carry in recreational greenspaces. We collected ticks bimonthly along trails and designated recreational areas in 17 publicly accessible greenspaces, in and around Gainesville, Florida, USA. We collected *Amblyomma americanum*, *Ixodes scapularis*, *Amblyomma maculatum*, *Dermacentor variabilis*, *Ixodes affinis*, and *Haemaphysalis leporispalustris*. Across the six tick species collected, we detected 18 species of bacteria or protozoa within the *Babesia*, *Borrelia*, *Cytauxzoon*, *Cryptoplasma (Allocryptoplasma)*, *Ehrlichia*, *Hepatozoon*, *Rickettsia*, and *Theileria* genera, including pathogens of medical or veterinary importance. While tick abundance and associated microorganism prevalence and richness were the greatest in natural habitats surrounded by forests, we found both ticks and pathogenic microorganisms in manicured groundcover. This relationship is important for public health and awareness, because it suggests that the probability of encountering an infected tick is measurable and substantial even on closely manicured turf or gravel, if the surrounding landcover is undeveloped. The presence of medically important ticks and pathogenic microorganisms in recreational greenspaces indicates that public education efforts regarding ticks and tick-borne diseases are warranted in this region of the United States.

## 1. Introduction

Greenspaces in urban environments are recognized to provide health benefits to residents and to conserve biodiversity and ecosystem functions [1,2,3,4]; however, city greenspaces can increase the risk of human and companion animal exposure to wildlife-associated ectoparasites, such as ticks and their associated pathogens [5,6]. Growing evidence suggests that urban environments pose a risk for exposure to ticks and tick-borne disease (TBD) agents, that was previously under-appreciated [6,7,8,9,10]. Tick vectors are expanding their geographical range [11] and are increasingly reported in urban greenspaces. In North America, this trend includes two tick species of medical and veterinary importance: *Ixodes scapularis* (Say) [9,10], and *Amblyomma americanum* (Linneaus) [7,8,12,13,14,15]. While it is clear that greenspaces pose a risk of exposure to ticks and TBD agents, it is less clear how this relates to specific landscape features or habitats [16]. A more detailed understanding of environmental features within and surrounding greenspaces that increase TBD risk will assist urban planners and land managers to manage habitat in a way that reduces risk, to increase public awareness in high-risk areas, and to design future greenspaces in ways that may reduce contact with tick vectors.

The southeastern USA has multiple tick species of medical or veterinary importance, and the rising emergence of less understood tick-borne microorganisms [17]. For example, Florida has multiple tick species of medical and veterinary importance [18,19] and reports in-state-acquired human TBD cases each year, such as ehrlichiosis, spotted fever group (SFG) rickettsioses, and to a lesser extent, Lyme disease [20]. The majority of ehrlichiosis and rickettsial diseases are reported from the north and central parts of the state [20], and underdiagnosis of TBD may underestimate the actual number of TBD cases [21,22,23].

Although greenspaces have been shown to pose a risk of exposure to TBD agents for humans and companion animals [7,8,24], there has been limited research on the influence of habitat structure or local landcover on ticks and tick-borne pathogens, particularly in the southeastern United States. Most work has concentrated on the urbanized fragmented forest ecosystems of the northeastern and Midwest United States [16], or urban greenspaces of Europe [25]. Our study aimed to define the impact of greenspace characteristics on the abundance and diversity of tick species and their associated microorganisms (bacterial and protozoal) at two spatial scales: the habitat within a greenspace, and the associated landscape surrounding the greenspace. We hypothesized that the habitat composition within greenspaces, whether it was natural habitat or manicured turf, would impact the abundance of ticks and prevalence of tick-borne disease agents. We also hypothesized that the local landscape surrounding greenspaces would influence tick abundance and tick-borne microorganism prevalence and diversity.

## 2. Materials and Methods

### 2.1. Experimental Design and Site Selection

**Figure 1:**
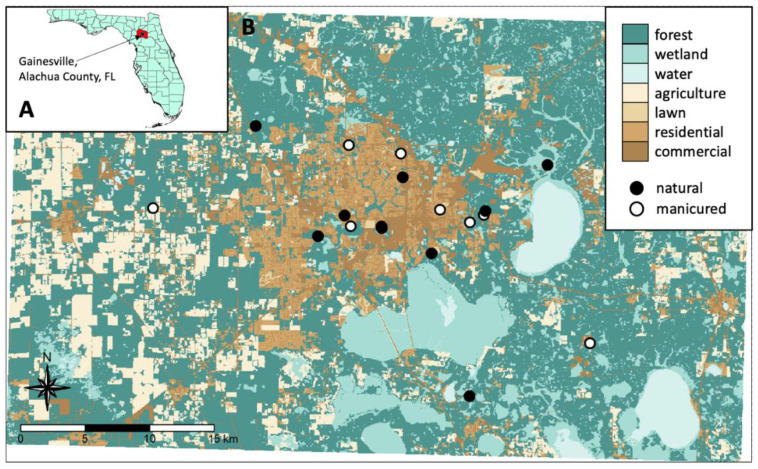
Landcover map showing (**A**) the State of Florida, USA, with Alachua County highlighted in red, and (**B**) the 17 greenspaces sampled in Gainesville, Florida, USA, and the surrounding area.

We collected ticks at 17 recreational greenspaces in and surrounding the Gainesville, Florida metropolitan area (latitude and longitude = 29.651634, −82.324829) during a six-month period of the year (January through June 2021), when *A. americanum* and *I. scapularis* were actively questing [18]. We chose to focus our efforts on one county, Alachua County (62 km^2^), because it has many publicly accessible greenspaces in the form of urban forests, wildlife management areas (WMAs), public gardens, and city, county, and state parks, all within the Gulf Coast Forest ecosystem of the southeastern USA, which includes pine forests, hardwood hammocks, hardwood swamps, mixed hardwood–pine forests, and wetlands. These greenspaces also provide a wide range of infrastructure for recreational activities, such as hiking trails, biking trails, horse trails, hunting areas, campgrounds, and picnic facilities, and reside in a patchwork alongside urban developments, such as housing and apartment complexes, shopping centers, and other human-built structures that are associated with human and companion animal activity (Figure 1). This region in north–central Florida has also been previously found to be suitable for medical and veterinary important tick species [26], and in-state-acquired TBD cases have been reported in humans since 2012 [20].

To assess how spatial scale influenced tick abundance and pathogen prevalence, we selected sampling sites based on (1) the habitat composition within a greenspace where we collected ticks, and (2) the habitat composition surrounding the greenspace. Beginning with a list of 36 publicly accessible candidate greenspaces within or closely surrounding the Gainesville metropolitan area, we categorized each greenspace as being composed of manicured habitat (defined as regularly managed turf or lawn, gardens, picnic areas, or cement/gravel roads), natural habitat (defined as unmodified forest or wetland areas), or both.

To quantify the amount of developed versus undeveloped landcover surrounding each candidate sampling site, we created an index of development. We utilized the publicly available Florida Land Cover Classification System (2018), which is a 10 m × 10 m resolution land cover dataset. We reduced the number of land cover classes by reclassifying the land cover map into seven representative categories for our study area (agriculture, commercial, forest, lawn or mowed grass, residential, water, and wetland; Supplementary Table S1). We further distilled the seven landcover types to a univariate descriptor using principal component analysis (PCA). Principal component analysis was conducted using the princomp function in the statistical package, R [27].

Within a greenspace, nine sites each represented either a manicured or natural habitat (Figure 1). Together, the nine sites within each habitat type varied across the development gradient surrounding the site. Overall, 18 sites in 17 different publicly accessible greenspaces were sampled in this study, as one greenspace included both manicured and natural sampling sites (Supplementary Table S2). For all recreational greenspaces, collecting permits for scientific sampling were obtained from respective land managing agencies (Supplementary Table S2).

### 2.2. Sampling Design and Field Methods

We visited each sampling site from the first week of January 2021 to the last week of June 2021. We sampled each site twice a month for a total of 12 sample periods for the entire project. At each sampling session per site, we collected ticks on ten 100 m transects (1 km total) that followed trails or designated recreational areas, in order to mimic human or domestic animal activity. Transects were chosen only in the designated habitat (manicured or natural) in each greenspace, and were confined to designated walking paths, trails, or lawns. We completed each 100 m transect in a continuous line, with the next transect beginning where the last one ended. If a greenspace had >1 km of trails or space within a specific habitat type, different transects were sampled randomly across time.

We used dragging and flagging methods for tick collection. We chose to use these sampling methods in our study because they mimic human and animal movement through the environment, and enable the estimation of the human and companion animal encounter rate with medical and veterinary important questing tick species [28,29]. We attached 1 m × 1 m white flannel cloths to dowel rods and used dragging to sweep the cloth on low-lying vegetation and the ground. Thus, sampling for 1 km using the 1 m^2^ cloth allowed us to estimate the density of ticks in 1 km^2^.

When bushes or tree limbs intersected our transect, we used flagging to sample the vertical vegetation. Ticks were collected every 10 m along transects and placed in 1.5 µL centrifuge tubes containing 75% ethanol. Adults and nymphs were stored individually. Larvae attached to the cloth were placed in one pool limited to a maximum of 50 individual larvae for that specific sampling site and sampling period.

We sampled for ticks between 10:00 and 16:00 h, waiting until after dew from the night before had evaporated. We did not attempt sampling if it had rained that day or was excessively windy. If weather conditions did not permit sampling on a scheduled sampling period, we visited the sampling site within the same week when weather conditions were more favorable.

### 2.3. Tick Identification and Storage

Ticks were stored at −20 °C prior to species identification and DNA extraction. Ticks were identified by life stage, sex, and species, using a Leica light microscope (Leica, Buffalo Grove, IL, USA) and taxonomic morphological or pictorial keys [30,31,32,33,34,35]. All ticks were placed on a frozen block (Corning™ XT Cooling Core Thermo Fisher Scientific, Carlsbad, CA, USA) during the identification process, to reduce DNA shearing that occurs during freeze–thaw cycles.

To confirm the species identity, we conducted molecular identification analysis of the *Haemaphysalis* specimen. DNA was extracted (described below) from a posterior leg, and amplified using conventional polymerase chain reaction (PCR), using primers (HCO2198/LCO1490) that targeted a 710 bp fragment of the cytochrome c oxidase subunit I (cox1) gene, following the conditions outlined in [36]. The resulting fragment was Sanger-sequenced in a commercial laboratory and the sequence identity was compared to sequences in GenBank.

### 2.4. DNA Extraction

We extracted DNA from tick specimens with the Qiagen DNeasy blood and tissue kit (Qiagen, Valencia, CA, USA). For three natural sampling sites that had a high abundance of *A. americanum*, we only extracted DNA from a subset of ticks. We calculated the size of the subset needed to estimate true prevalence for pathogens of *A. americanum*, using EPI tools (https://epitools.ausvet.com.au/prevalencess, accessed on 15 December 2022). From these three sites, we selected an equal number of ticks from each sampling period, sampling session (month), life stage (adult/nymph), and sex.

To start the extraction process, we bisected each adult and nymphal tick medially on a piece of parafilm with a flame sterilized scalpel. Both halves of the ticks were then placed in a 1.5 mL microcentrifuge tube containing 180 µL ATL buffer and 20 µL Proteinase K. Larval pools were first homogenized with high-density zirconium oxide beads, using a Mini Bead Beater 16 (GlenMills, Clifton, NJ, USA) and 0.1 M PBS. Supernatant was centrifuged and 160 µL of the solution was placed in the ATL buffer and proteinase K solution. Samples were incubated at 56 °C for 24 h. After digestion was completed, we followed the manufacturer’s protocol. DNA quality and quantity were measured for each sample or larval pool (Nanodrop One, Thermo Fisher Scientific). DNA was stored at −20 °C until PCR was performed.

### 2.5. Polymerase Chain Reaction, Gel Electrophoresis, and Sequencing

Polymerase chain reaction was used to detect tick-borne bacterial or protozoan taxa of relevance to public and veterinary health. Our PCR assays screened for a broad range of bacterial genera (*Anaplasma*, *Borrelia*, *Ehrlichia*, and *Rickettsia*), as well as piroplasms in the phylum Apicomplexa (*Babesia*, *Hepatozoon*, *Cytauxzoon*, and *Theileria*). Extracted tick samples were screened using multiple polymerase chain reaction methods: conventional (PCR), nested (n-PCR), hemi-nested (h-PCR), and real-time quantitative (q-PCR) PCRs.

For all the tick species collected, we used two assays to detect the spotted fever group (SFG) *Rickettsia* spp.: a PCR that targeted the *ompA* gene [19,37] and a qPCR that targeted the *17kDa* antigen gene [38,39,40]. Positive samples from *ompA* and *17kDa* PCRs were run through an additional h-PCR assay which excluded *Rickettsia amblyommatis*, yet targeted other low-abundant pathogenic SFG *Rickettsia* spp. co-infections by amplifying the *phospholipase D family protein* gene (*PLA*) [41].

To detect Apicomplexa parasites, we amplified the *18s rRNA* gene [40,42], and conduced an n-PCR, targeting the *flagellin B (flab)* gene for *Borrelia* spp. within the *Borrelia burgdorferi* sensu lato complex [19,43]. Two additional n-PCRs targeting *Borrelia* spp., including relapsing fever *Borrelia* spp., one amplifying the *ospA* gene and one amplifying the *intergenic spacer* (*IGS*) gene, were run [44,45] for every tick species, except *A. americanum*.

We screened all samples for Anaplasmataceae (*Ehrlichia* and *Anaplasma* spp.) with n-PCR, targeting the *groEL* gene [19,46]. To confirm the identification of microorganisms to genus, positive samples were amplified with a broad *Rickettsia* spp. primer set, amplifying the *gltA* gene [45,47], and a broad Anaplasmataceae primer set, amplifying the *16s* gene [40,48]. *Ehrlichia*-positive samples were also run through a species-specific n-PCR, targeting the *gltA* gene of the Panola Mountain Ehrlichia species [49].

An Eppendorf Master cycle pro S (Eppendorf, Hamburg, Germany) was used for each PCR, h-PCR, and n-PCR assay, while an ABI 7500 Fast Real-Time PCR System Machine (Thermofisher, Waltham, MA, USA) was used for qPCRs. All PCR assays were conducted using a maximum of 50 ng/µL DNA. Each assay contained a negative template control using H_2_O, and a positive control using either synthetic constructs or cloned products from previous positive samples with a bacteria/protozoan species targeted by each PCR assay. We conducted gel electrophoresis on all PCR, h-PCR, and n-PCR products, running a 1.5–2% agarose gel with the Red View DNA Gel Stain (Genecopoeia, Rockville, MD, USA). All positive samples were mailed to Functional Biosciences (Madison, WI, USA) or Eurofins Genomics (Louisville, KY, USA) for Sanger sequencing, with the appropriate primer set for each gene amplified. We used Geneious Prime (Biomatters, New Zealand) to align sequences, and then NCBI BLAST (http://www.ncbi.nlmn.nih.gov/BLAST, accessed 20 December 2022) to compare results with previously published sequences. To identify a bacteria or protozoan to species, all aligned sequences were matched with those within NCBI BLAST at a 99% sequence similarity or higher. If sequences were below <99% identity, microorganisms were reported to the genus only.

### 2.6. Tick Density and Abundance Analysis

Mean tick abundance and density per 1 km^2^ were calculated per tick species and life stage by taking the average across the 12 sampling time periods and then across sampling sites in each habitat type. We used generalized linear models (GLMs) to test the effects of the greenspace habitat type (manicured or natural) and the influence of surrounding development on the total abundance of *A. americanum* and *I. scapularis*. To determine the most predictive local landscape buffer size that was the most predictive for abundance of *A. americanum* and *I. scapularis*, we quantified the development gradient at 500 m, 1 km, 2 km, 3 km, and 4 km radii [50,51]. We ran univariate generalized linear models with a Poisson distribution for all buffer sizes. We chose the most predictive buffer size for each species, using the Akaike Information Criterion (AIC) scores, and retained the buffer size with the lowest AIC for future analyses (Supplemental Table S6).

We tested the effect of habitat type and development gradient on response variables using GLMs with a negative binomial distribution, considering all possible combinations of explanatory variables. The top models were selected as the model with the smallest AIC or within two delta AIC of the top model. Model residuals were visually inspected for model assumptions. We tested spatial autocorrelation in the model residuals using Global Moran’s I and two spatial weights matrices: one using a Gabriel graph to define neighbors [52], and one using inverse distance weights.

### 2.7. Infected Tick Microorganism and Pathogen Analysis

To calculate the prevalence of microorganism species for each tick species, the number of ticks positive for each microorganism was divided by the total number of ticks of that species tested. For larval pools, both percentage positive (# of positive pools/# total pools) and minimum infection rate (# of positive pools/# of total larvae examined) were calculated. A Wilson 95% CI was calculated using EPI tools (https://epitools.ausvet.com.au/ciproportion, accessed 15 December 2022) to calculate confidence limits for a sample proportion for the apparent prevalence of microorganisms identified in each tick species and larval pool tested. If present, co-infections for individual adult or nymphal ticks were also recorded.

We used generalized linear models (GLMs) with a negative binomial distribution to test the effects of habitat type (natural versus manicured) and the influence of surrounding development on (1) the presence of infected ticks (presence was defined as encountering at least one adult or nymph tick of *A. americanum* or *I. scapularis* infected with a confirmed or possibly pathogenic bacterium or protozoan during the total sampling period), (2) the abundance of infected ticks over the total sampling period, and (3) the richness of pathogenic bacteria or protozoa. We classified a microorganism as pathogenic if they were associated with clinical illness in either humans or companion animals. To determine which habitat factors influenced pathogen dynamics, we used GLMs with a binomial distribution for the presence of infected ticks and a negative binominal distribution for abundance of infected ticks and pathogen richness. We considered all possible combinations of explanatory variables, and the top model was selected as the model with the smallest AIC or within two delta AIC of the top model. Model residuals were visually inspected for model assumptions.

All analyses were conducted in R Ver. 4.1.2 [27], using the ‘MASS’ packageVer. 7.3-58.1 for GLMs with a negative binomial distribution [53], using the ‘spdep’ package Ver.1.2-7 for calculating distance matrices and Global Moran’s I tests [54], using the ‘MuMIn’ package Version 1.43.17 [55], for model comparisons. The ‘ggplot2’ package Version 3.3.6 was used for data visualization [56].

## 3. Results

### 3.1. Tick Collections and Density Measurements

**Figure 2:**
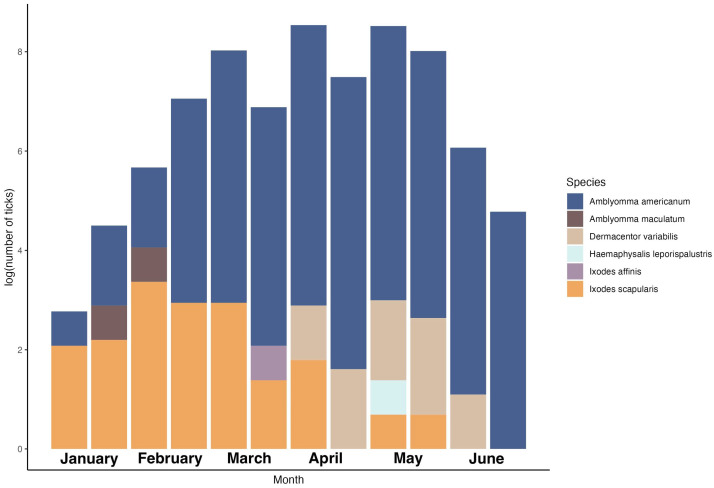
Total number of ticks (log-transformed) by species collected in 17 greenspaces in Alachua County, Florida, USA, per month (two sampling sessions per month), from January to July 2021.

From the first week of January 2021 to the last week of June 2021, we collected six tick species: *Amblyomma americanum*, *Amblyomma maculatum* Koch, *Dermacentor variabilis* (Say), *Haemaphysalis leporispalustris* (Packard), *Ixodes affinis* Neumann, and *I. scapularis* of varying life stages (Table 1, Figure 2). Most of the ticks collected were *A. americanum nymphs* (*n* = 1042) and adults (*n* = 673), and this was the only species for which all three life stages were collected. Overall, we collected 21 pools (*n* = 940 individuals) of *A. americanum* larvae. One pool contained 36 individual larvae, two pools contained two larvae, and 18 pools contained 50 larvae each. In addition, we collected 89 *I. scapularis* adults, 18 *D. variabilis* adults, two *A. maculatum* nymphs, one *H. leporispalustris* nymph, and one *I. affinis* adult. We collected ticks at 17 of the 18 sampling sites during the study (Supplementary Figure S2; Supplementary Table S3).

### 3.2. Tick-Associated Bacterial and Protozoal Microorganisms

From the analyzed samples, we detected 18 species of bacteria or protozoa across eight genera: *Babesia*, *Borrelia*, *Cryptoplasma* (*Allocryptoplasma*), *Cytauxzoon*, *Ehrlichia*, *Hepatozoon*, *Rickettsia*, and *Theileria* (Table 2 and Table 3). All unique sequences generated in this study were assigned an NCBI GenBank accession number (Supplemental Table S4). Larval *A. americanum* pools tested positive for three main bacterial organisms: *Borrelia lonestari*, *R. amblyommatis*, and *R. parkeri* (Table 3). No pools tested positive for Anaplasmataceae or Apicomplexa species. One larval pool was positive for both *B. lonestari* and *R. amblyommatis*, and both larval pools that were positive for *R. parkeri* were also positive for *R. amblyommatis*.

Overall, tick-borne microorganisms were distributed across Alachua County greenspaces and within both natural and manicured habitat types (Supplementary Table S2). While most ticks infected with pathogenic *Ehrlichia* were found in natural habitat within greenspaces, one of the 23 *Ehrlichia*-infected ticks (ticks co-infected with two *Ehrlichia* spp. are only counted once as one entity) was found in a manicured habitat. The human pathogen, *R. parkeri*, was found in all three life stages of *A. americanum*. Of the three *A. americanum* adults or nymphs that were positive for *R. parkeri*, two were found in the manicured habitat. Both positive larval pools for *R. parkeri* were from the same sampling site as the one adult infected with *R. parkeri*. These ticks were collected in the natural habitat. Lastly, one of eight *I. scapularis* ticks positive for *Babesia odocoilei* was found in a manicured habitat.

Nearly 7% of adult and nymphal *A. americanum* and adult *I. scapularis* were co-infected with two bacterial or protozoal microorganisms (Table 4). In *A. americanum* adults and nymphs, there were a total of eleven unique combinations of microorganisms in ticks with dual infections, and six unique combinations of microorganisms in ticks with triple infections (Supplementary Figure S3). Most dual and all triple infections included *R. amblyommatis*. In *I. scapularis* adults, there were four different combinations of microorganisms in ticks with dual infections (Supplementary Figure S3).

### 3.3. Effect of Habitat Type and Landcover on Tick Abundance and Pathogen Infection

PCA analysis of percent habitat within a 1 km buffer of the 18 sampling sites revealed that the first principal component represented a human development gradient. This developed-to-undeveloped axis explained 69% of the variance in land cover class variables and had a strong negative relationship with commercial and residential cover classes, and a strong positive relationship with forest cover classes (Supplementary Figure S1).

**Figure 3:**
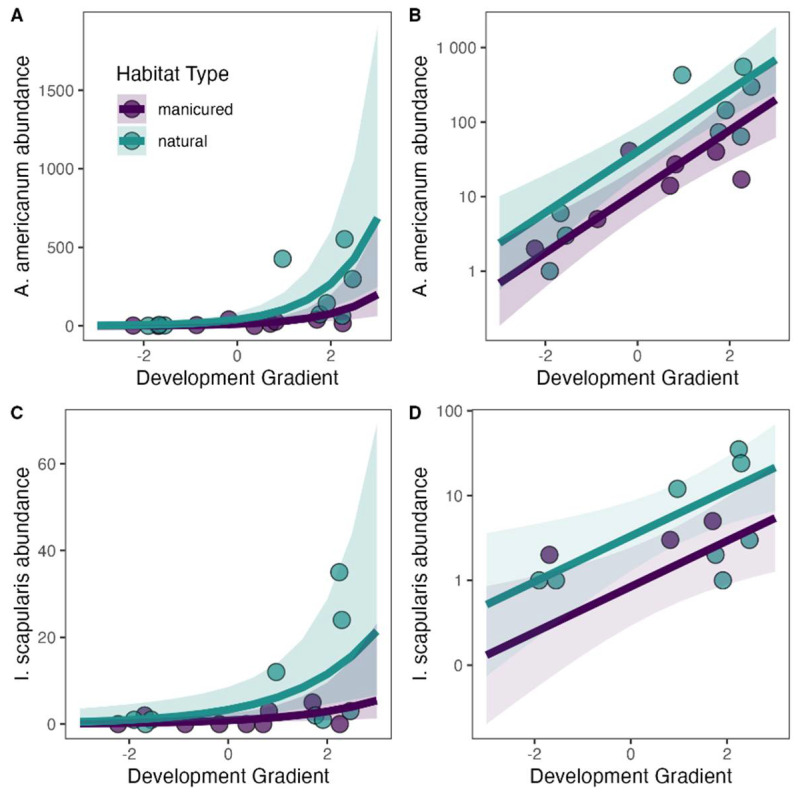
Model predictions for the influence of the development gradient (developed to undeveloped) and habitat type (green = natural; purple = manicured) on raw (**A**,**C**) or log-transformed (**B**,**D**) tick abundance for *Amblyomma americanum* adults and nymphs combined (**A**,**B**), and *Ixodes scapularis* adults (**C**,**D**) in greenspaces in Alachua County, Florida, USA. Lines indicate model prediction and grey regions represent the 95% CI of the prediction.

Both environmental scales, habitat type within the greenspace (natural or manicured) and the local landscape surrounding the greenspace (the development gradient) had significant effects on the abundance of *A. americanum* and *I. scapularis* (Table 5, Supplementary Table S5). We found significantly more *A. americanum* and *I. scapularis* in natural habitat than in manicured habitats within greenspaces. We also found differences in the abundance of *A. americanum* and *I. scapularis* along a development gradient that surrounded the greenspaces: we observed a higher tick abundance in sites surrounded by less development and more forest (Figure 3) than in more developed areas. A buffer size of 500 m was the best fit to estimate the scale of influence of the development gradient on the abundance of both tick species (Supplementary Table S6). We observed no spatial autocorrelation in model residuals (Global Moran’s I > 0.05) across all top models.

**Figure 4:**
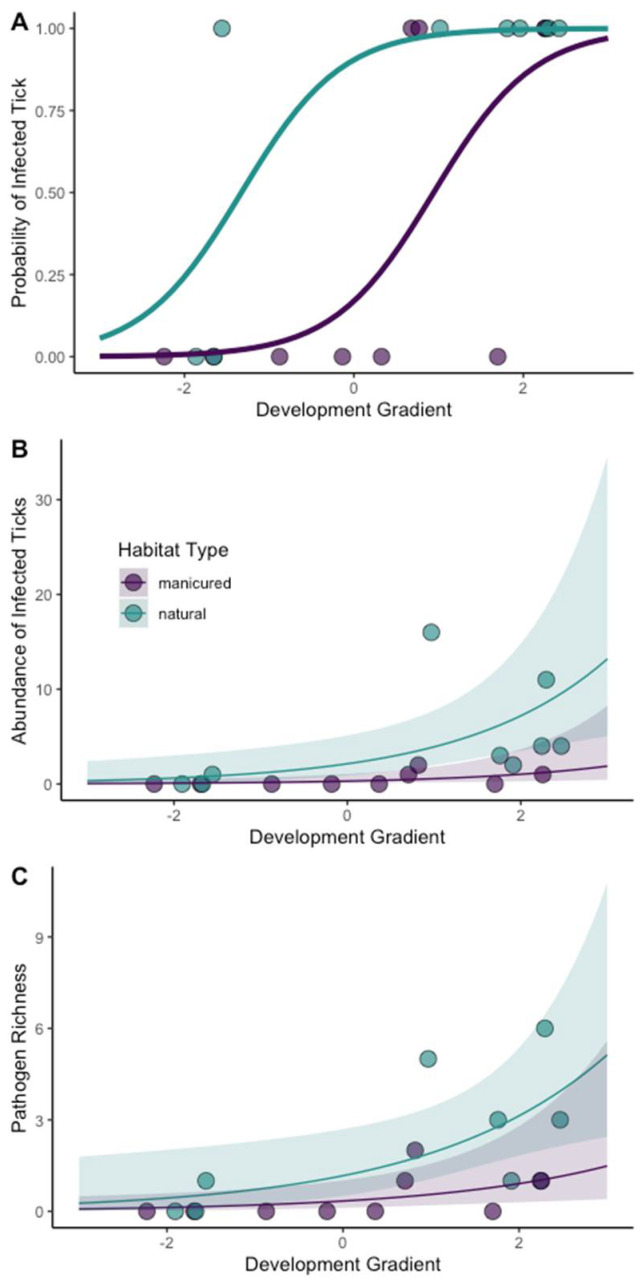
Model predictions for (**A**) the probability of encountering an infected tick based on the presence or absence of an infected tick, (**B**) the abundance of infected ticks, and (**C**) pathogen richness along the development gradient in greenspaces in Alachua County, Florida, USA. These analyses included all tick species and microorganisms considered to be pathogens of medical or veterinary importance. The human development gradient was derived from a principal component of landcover proportions from within 500 m radii of each site. Negative values on the development gradient represented a more human-developed landscape and positive values represented less developed landscapes that had greater amounts of native habitats.

Both greenspace habitat type (natural or manicured) and the development gradient had significant relationships with the presence of an infected tick, the abundance of infected ticks, and pathogen richness (Table 5; Figure 4). An infected tick was more likely to be encountered at natural sites than manicured sites and sites surrounded by less development, regardless of habitat type. We also found the abundance of infected ticks and pathogen richness to be significantly greater in natural sites than manicured sites. Both measures also significantly increased as the forest habitat surrounding the site increased (Supplementary Table S7).

## 4. Discussion

Our sampling effort occurred along walking trails and other areas of high human traffic. Combined with dragging and flagging, this sampling effort mimicked human use of greenspaces. We found that undeveloped landcover in the surrounding landscape and natural habitat within greenspaces had strong positive effects on tick density and the probability of encountering an infected tick while walking in greenspaces. Our results suggest that even in manicured turf and landscaping, infected ticks occurred along walking trails and paths, particularly when those manicured habitats were surrounded by moderate amounts of undeveloped landcover (Figure 4A). Overall, sampling sites, ticks, and tick-borne pathogens were readily encountered during the six-month period of the survey.

### 4.1. Tick Abundance

All tick species collected in this study can vector disease agents to humans and their companion animals. The two most abundant species of ticks in this study, *A. americanum* and *I. scapularis*, were present in most surveyed greenspaces. We detected *D. variabilis* infrequently and *H. leporispalustris* once during our survey. Although *H. leporispalustris* rarely feeds on humans [66], its collection on a drag or flag in our study shows that humans and companion animals may encounter this species in a recreational greenspace. *Ixodes affinis*, a species morphologically similar to *I. scapularis*, feeds on a wide variety of mammals, including companion animals and humans [67], and is considered an enzootic vector in the maintenance of *B. burgdorferi* sensu stricto [68,69,70,71].

### 4.2. Tick-Borne Bacteria and Protozoa

We found microorganisms of medical importance in two out of the six species of ticks collected during this study (*A. americanum* and *I. scapularis*). We recorded the human pathogen, *R. parkeri*, in all three stages of *A. americanum*. Although at a low prevalence (Table 2), finding *R. parkeri* is significant, as it is a causative agent of Rickettsiosis, a disease that can cause mild to, sometimes, severe clinical symptoms in humans [62,72,73,74]. We found this pathogen in ticks collected from natural and manicured greenspaces. Questing *A. americanum* adults and nymphs in the USA, including Florida, have shown to be infected with *R. parkeri*, but at a low prevalence [19,41,75,76]. The main vector for *R. parkeri* is *A. maculatum*, which we collected at two sampling sites in our study. Although none of the *A. maculatum* in this study tested positive for *R. parkeri*, our pilot data, at one of our sampled areas from Alachua County in 2019, did contain an *A. maculatum* positive for *R. parkeri* [Accession #: MT273010]. *Amblyomma americanum* may become infected with *R. parkeri* when co-feeding with *A. maculatum* on reservoir hosts [77].

To our knowledge, our study may have the first record of *R. parkeri* in the larval stage of wild-caught questing *A. americanum* in the USA, adding to the rising importance of this tick species in the spread of this SFG *Rickettsia* spp. Transstadial and transovarial transmission of *R. parkeri* in *A. americanum* has been demonstrated in a laboratory setting [78], and our study suggests that transovarial transmission also occurs in situ. The presence of *R. parkeri* in *A. americanum* collected at recreational greenspaces in the area poses a transmission risk of the pathogenic microorganism to humans and affirms the possibility of contracting SFG rickettsiosis. Pathogenic *R. parkeri* in recreational greenspaces (both in manicured and natural habitats) substantiate the risk of infection with SFG *Rickettsia* spp. in the southeastern US.

We detected three *Ehrlichia* species in *A. americanum*: *E. chaffeensis*, *E. ewingii*, and the Panola Mountain *Ehrlichia* sp. Both adults and nymphs contained pathogenic *Ehrlichia* species, but only four nymphs were infected, with three of them being infected with the Panola Mountain species. Both *E. chaffeensis* and *E. ewingii* are pathogenic to humans and companion animals, and are the causative agents for granulocytic ehrlichiosis and monocytic ehrlichiosis, respectively [59,79]. The Panola Mountain *Ehrlichia* sp. is an emerging pathogenic *Ehrlichia* species implicated in human and domestic-animal cases (dog and goat) of ehrlichiosis [49,60,61,80]. Although more abundant in natural habitat within greenspaces, our study indicates the presence of pathogenic *Ehrlichia* species in manicured greenspaces in Alachua County. We found a low, but persistent, prevalence of *Ehrlichia* spp. within our study, as we detected *Ehrlichia* in 5 out of 6 months. This consistency indicates that recreational greenspaces pose a risk for both human and animal health.

We detected *B. odocoilei*-infected *I. scapularis* in north–central Florida. *Babesia odocoilei* was recently found in two humans presenting clinical babesiosis symptoms from Canada, possibly indicating its role as an emerging tick-borne pathogen and cause of human babesiosis in North America [64]. *Ixodes scapularis* is the only confirmed vector for *B. odocoilei*, and the agent has been found in questing or even engorged ticks on dogs and domestic cats across North America [64,81,82,83,84,85]. Our report adds *B. odocoilei* to the list of potentially infectious agents found in *I. scapularis* in Florida. Due to the detection of this parasite in questing *I. scapularis* ticks at recreational greenspaces, there may be a risk for the microorganism to infect humans and companion animals, if bitten by the tick species. Overall, *B. odocoilei* has been shown to cross react in serological tests of *B. duncani*, a known causative agent for human babesiosis [64]. Thus, healthcare professionals should be aware of other *Babesia* species in the area, which could be of medical concern and confound laboratory tests.

In addition to detecting many tick-borne microorganisms of human health importance, we found several of animal health concern belonging to the protozoan phylum Apicomplexa (*Babesia*, *Cytauxzoon*, and *Theileria* spp.) in questing *A. americanum*, *I. scapularis*, and *D. variabilis*. Microorganism species such as *B. odocoilei*, *Babesia sp.* (Coco), *Cytauxzoon felis*, and *Theileria cervi*, have all been reported to cause clinical symptoms in domestic animals or wildlife, adding to their importance in the veterinary field [63,64,86,87]. Naturally infecting white-tailed deer (*Odocoileus virginianus*) and historically described as non-pathogenic, *B. odocoilei* has now been implicated to cause clinical babesiosis symptoms or mortality in susceptible wild or captive cervid populations in North America [65,88,89,90,91]. *Babesia* species (Coco) has been identified in domestic dogs and other animals, where it has been shown to cause clinical signs of thrombocytopenia and anemia in immunocompromised or splenectomized canines [57]. *Cytauxzoon felis* is the causative agent for cytauxzoonosis (also known as bobcat fever), an emerging hemoprotozoal pathogen in domestic cats, which may cause subclinical-to-fatal disease [58,92,93,94]. Although less likely to cause clinical symptoms, *T. cervi* has been reported in deer populations, including mortality in fawns [63,95]. Overall, finding the presence of pathogenic Apicomplexa species in questing *A. americanum*, *I. scapularis*, and *D. variabilis* ticks poses additional TBD risks for companion animals and wildlife that may frequent or inhabit recreational greenspaces in Alachua County.

In addition to human and animal pathogens, we found that five of the six tick species harbored many tick-borne bacteria or protozoa of unknown pathogenicity, or that are undescribed and could still be of emerging medical or veterinary importance (Table 2). The detection of a wide range of microorganisms suggests future efforts in understanding the tick-microbiome and tick-host dynamics are needed.

We detected co-infections (dual or triple) between bacteria and/or protozoa in *A. americanum* and *I. scapularis*. Co-infections can impact pathogen transmission, severity and morbidity in animals, and impact tick biology or evolution [96,97]. Most co-infections were in *A. americanum* with the bacteria *R. amblyommatis* or the protozoa *T. cervi*, which were present in most dual infections and all triple infections. Some *A. americanum* infected with *Ehrlichia* species were either dual- or triple-infected with *R. amblyommatis*. Although bacterial co-infections with both *Rickettsia* and *Ehrlichia* spp. In humans have not been detected, co-infections in *A. americanum* have been described [8,98]. We also detected co-infections of the Panola Mountain *Ehrlichia* sp. with both *E. chaffeensis* and *E. ewingii* in adult *A. americanum*, indicating that there may be a risk of human and animal contact between two pathogenic *Ehrlichia* species if bitten by a co-infected tick. Co-infections *A. americanum* have been observed in a prior study [49].

The high abundance of *R. amblyommatis* in *A. americanum* may limit the detection of *R. parkeri* and other common pathogenic SFG *Rickettsia* species within an individual tick because of competitive exclusion in common diagnostic assays. These assays have difficulty identifying these less abundant, but medically relevant, pathogens when there is a co-infection with *R. amblyommatis* [41,77]. Recently, a cost-effective hemi-nested PCR assay, was developed to overcome the occurrence of false negatives for *R. parkeri* in vectors that are co-infected with *R. amblyommatis* [41]. Our study utilized this assay and identified the presence of *R. parkeri* in three *A. americanum* co-infected with *R. amblyommatis*, which would have otherwise been overlooked by other diagnostic assays. Overall, the detection of co-infections in our ticks adds to the complexity of the tick pathogen microbiome and suggests the need for continuing research on the importance of co-infections for both human and animal health.

### 4.3. Effect of Human Development on Ticks and Tick-Borne Microorganisms

Tick species richness and tick abundance varied greatly across our study area, as did the richness of tick-borne pathogens and abundance of infected ticks. While we found the highest diversity and abundance of ticks and pathogens in natural habitat within greenspaces, we also found a substantial subset in manicured habitats including turf lawn, picnic areas, or along paved pathways. The presence of infected ticks in multiple manicured greenspaces throughout the region suggests the environmental conditions in these spaces may be sufficient for tick population establishment, persistence, and infection with pathogenic microorganisms.

Our study suggests that many recreational greenspaces within the Gainesville metropolitan area have the potential to sustain infected tick populations. Overall, the abundance of *A. americanum* and *I. scapularis*, the abundance of infected ticks, and pathogen richness (i.e., more infected ticks and greater pathogen richness in less developed areas) was positively associated with increasing forest cover. The only greenspace in which we did not collect any ticks was a manicured site located behind a residential neighborhood in a live oak hammock forest.

The landscape composition surrounding a greenspace influences tick population establishment and persistence, because it affects the availability of wildlife hosts [6]. As ticks move only small distances during each life stage, ticks rely on hosts movement for dispersal, which in turn determines distribution patterns [99]. Once ticks arrive in a novel habitat, their survival and persistence are dependent on sufficient vegetation and microclimatic conditions (mainly humidity) to prevent desiccation and the occurrence of hosts to sustain tick [100]. The degree to which wildlife utilizes and moves throughout our study area is unknown, but the high amounts and connectivity of undeveloped landcovers likely facilitated the observed high tick density in greenspaces surrounded by forest cover [101].

The present study was limited by several factors. Our sampling occurred only during the first half of the year, and thus does not fully represent the diversity of tick life stages nor demographic changes that occur throughout the year. Our data are also only representative of one county in Florida, and thus interpretation over broad spatial scales is not warranted. Nonetheless, our data indicate that landscape scale differences in habitat can drive pathogen presence, and that medically and veterinary important ticks do occur in urban recreational areas in the Gulf Coast Forest ecosystem in the southeastern US.

## 5. Conclusions

The habitat composition of urban recreational greenspaces and composition of the surrounding local landscape has direct implications for tick and TBD risk. These findings are relevant, because recreational greenspaces in urban areas are a cultural and social resource. Initiatives to increase the number of greenspaces have distinct benefits for human well-being, wildlife conservation, and climate change mitigation [102]. In conjunction with these efforts, our findings highlight the need to understand the drivers of infected tick distributions in recreational greenspaces. Our study describes the hazard of a tick bite and subsequent TBD in 17 recreational greenspaces, and highlights how habitat management and public education within and surrounding these greenspaces could mitigate this hazard.

## Figures and Tables

**Table 1:** Average density per 1 km^2^ with standard error (SE) of nymphal and adult ticks collected by species, across each sampling bout from the first week of January 2021 to the last week of June 2021 and across location. Estimates are provided for two sampling site categories (9 natural sites and 9 manicured sites) in 17 greenspaces in Alachua County, Florida, USA. Zero indicates no ticks were collected.

Tick Species and Life Stage	Habitat Type	
	Natural (Mean ± SE)	Manicured (Mean ± SE)
*Amblyomma americanum* adult	5.82 ± 2.24	0.41 ± 0.12
*Amblyomma americanum* nymph	8.70 ± 3.47	0.94 ± 0.37
*Amblyomma maculatum* nymph	0.01 ± 0.01	0.01 ± 0.01
*Dermacentor variabilis* adult	0.15 ± 0.11	0.02 ± 0.01
*Haemaphysalis leporispalustris* nymph	0.01 ± 0.01	0
*Ixodes affinis* adult	0.01 ± 0.01	0
*Ixodes scapularis* adult	0.73 ± 0.35	0.09 ± 0.05

**Table 2:** Microorganism prevalence in individual nymphal and/or adult ticks collected from January 2021 to June 2021 across 17 greenspaces, in Alachua County, Florida, USA, with Wilson 95% CI and gene information retrieved. A reference is given to distinguish microorganisms of potential or known medical or veterinary importance, or if microorganisms were cited in human and domestic-animal case reports. The abundance of ticks that includes the referenced microorganisms was used as a response variable in the generalized linear model. It is also noted if a microorganism is of unknown pathogenicity.

Tick Species and Life Stage	Microorganism	n. Positive/n. Tested (%)	Wilson 95% CI	Gene Tested	Reference(s) to Medical or Veterinary Importance
*Amblyomma americanum* adult/nymph	*Babesia* sp.	16/1076 (1.5%)	0.9–2.4%	*18s*	Unknown pathogenicity
	*Babesia* sp. *(Coco)*	7/1076 (0.7%)	0.3–1.3%	*18s*	[57]
	*Borrelia lonestari*	14/1076 (1.3%)	0.8–2.2%	*flaB*	Unknown pathogenicity
	*Cytauxzoon felis*	1/1076 (0.1%)	0.0–0.5%	*18s*	[58]
	*Ehrlichia ewingii*	10/1076 (0.9%)	0.5–1.7%	*groEL*	[59]
	*Ehrlichia chaffeensis*	7/1076 (0.6%)	0.3–1.3%	*groEL*	[59]
	Panola Mountain *Ehrlichia* sp.	9/1076 (0.8%)	0.4–1.6%	*groEL*; *gltA*	[60,61]
	*Hepatozoon* sp A.	19/1076 (1.8%)	1.1–2.7%	*18s*	Unknown pathogenicity
	*Rickettsia amblyommatis*	583/1076 (54.2%)	51.2–57.1%	*ompA*; *17kda*	Unknown pathogenicity
	*Rickettsia parkeri*	3/1076 (0.3%)	0.1–0.8%	*PLA*; *17kda*	[62]
	*Theileria cervi*	48/1076 (4.5%)	3.4–5.9%	*18s*	[63]
*Amblyomma maculatum* nymph	*Rickettsia andeanae*	1/2 (50.0%)	9.5–90.5%	*ompA*; *gltA*	Unknown pathogenicity
	*Borrelia* spp., Anaplasmataceae spp. Apicomplexa spp.	0/2 (0.0%)	NA	*flaB*; *ospA*; *IGS*; *groEL*; *18s*	
*Dermacentor variabilis* adult	*Rickettsia rhiphicephali*	2/18 (11.1%)	3.1–32.8%	*ompA*; *gltA*; *PLA*	Unknown pathogenicity
	*Theileria cervi*	1/18 (5.6%)	1.0–25.8%	*18s*	[63]
	*Borrelia* spp., Anaplasmataceae spp.	0/18 (0.0%)	NA	*flaB*; *ospA*; *IGS*; *groEL*	
*Haemaphysalis leporispalustris* nymph	*Rickettsia* spp., *Borrelia* spp., Anaplasmataceae spp., Apicomplexa spp.	0/1 (0.0%)	NA	*ompA*; *flaB*; *ospA*; *IGS*; *groEL*; *18s*	
*Ixodes affinis* adult	*Rickettsia* sp.	1/1 (100.0%)	20.6–100.0%	*ompA*; *gltA*	Unknown pathogenicity
	*Borrelia* spp., Anaplasmataceae spp. Apicomplexa spp.	0/1 (0.0%)	NA	*flaB*; *ospA*; *IGS*; *groEL*; *18s*	
*Ixodes scapularis* adult	*Babesia odocoilei*	8/89 (9.0%)	4.6–16.7%	*18s*	[64,65]
	*Cryptoplasma (Allocryptoplasma)* sp.	13/89 (14.6%)	8.7–23.4%	*groEL*; *16s*	Unknown pathogenicity
	*Hepatozoon* sp. B.	1/89 (1.1%)	0.2–6.1%	*18s*	Unknown pathogenicity
	*Rickettsia* spp. endosymbiont	28/89 (32.5%)	22.8–41.7%	*ompA*	Unknown pathogenicity
	*Borrelia* spp.	0/89 (0.0%)	NA	*flaB*, *ospA*, *IGS*	

**Table 3:** Microorganism prevalence in 21 *Amblyomma americanum* larval pools collected in greenspaces in Alachua County, Florida, USA.

Microorganism	(n. Positive Pools/Total Pools) % Positives [Wilson 95% CI]	(n. Positive/Total Larvae) Minimum Infection Rate [Wilson 95% CI]
*Borrelia lonestari*	(2/21) 9.5% [2.7–28.9%]	(2/940) 0.2% [0.1–0.8%]
*Rickettsia amblyommatis*	(14/21) 66.6% [45.4–82.8%]	(14/940) 1.5% [0.9–2.5%]
*Rickettsia parkeri*	(2/21) 9.5% [2.6–28.9%]	(2/940) 0.2% [0.1–0.8%]

**Table 4:** The number of dual and triple infections in different life stages of Ixodid ticks collected in greenspaces in Alachua County, Florida, USA.

Species	Life Stage	n. of Triple Infections	n. of Dual Infection	n. of Single Infections	n. Tested
*Amblyomma americanum*	adult	6	40	292	498
*Amblyomma americanum*	nymph	0	31	266	578
*Amblyomma. maculatum*	nymph	0	0	1	2
*Dermacentor variabilis*	adult	0	0	3	18
*Ixodes affinis*	adult	0	0	1	1
*Ixodes scapularis*	adult	0	6	38	89

**Table 5:** Result of top GLM models evaluating the influence of landscape variables on tick abundance, infected tick presence and abundance, and pathogen richness for greenspaces in Alachua County, Florida, USA. Infected ticks were defined as nymphs or adults of any species of tick having a microorganism that was confirmed or potentially pathogenic (Table 2). The top GLMs (lowest AIC) for (a) *Amblyomma americanum* abundance, (b) *Ixodes scapularis* abundance, (c) presence of infected ticks of either species, (d) abundance of all ticks, and (c) pathogen richness are reported. The development gradient represented the developed-to-natural landcover gradient, using 500 m buffers for each site. The standard error for each estimate is indicated in parentheses. Complete model results are presented in Supplementary Tables S4 and S6. *** indicates *p* < 0.01; ** indicates *p* < 0.05; * indicates *p* < 0.1.

Species	Intercept	Habitat Type	Development Gradient	Pseudo R^2^
(a) *Amblyomma americanum* abundance	2.461 *** (0.377)	1.238 *** (0.525)	0.945 *** (0.165)	62.1
(b) *Ixodes scapularis* abundance	−0.169 (0.531)	1.371 ** (0.666)	0.620 *** (0.216)	49.0
(c) presence of infected tick	−1.591 (1.198)	3.835 (2.702)	1.690 * (0.971)	54.9
(d) abundance of infected ticks	−1.187 * (0.625)	1.945 *** (0.688)	0.608 *** (0.211)	61.0
(e) pathogen richness	−1.082 ** (0.550)	1.238 ** (0.600)	0.493 ** (0.197)	54.4

## Data Availability

The data presented in this study are available in the Supplementary Materials and sequence data is published in NCBI GenBank.

## Supplementary Materials

The following supporting information can be downloaded at: https://www.mdpi.com/article/10.3390/microorganisms11030756/s1, Table S1: Reclassification of Florida Land Cover Classification System. The Class ID and Class were obtained from the original Florida Land Cover Classification System (2018). Reclass and Reclass ID represent the land cover reclassification used for this study; Figure S1: PCA biplot of all sampling sites considered in this study. PC1, which represents nearly 70% of variation in landcover composition surrounding sites using a 1 km buffer, represents a developed (negative PC1 values)-to-undeveloped (positive values of PC1) gradient. Sites sampled in this study are represented in dark purple and dark green; Table S2: List of recreational greenspaces sampled in Alachua Co., Florida, with sampling site habitat type designation (natural or manicured), number of ticks collected, species of ticks identified, and microorganisms present at each site; Figure S2: Total tick abundance (log-transformed) by species collected across all 18 sampling sites. First 9 sites are designated as manicured, while the last 9 sites are designated as natural sites; Table S3: Total number of tick species collected per life stage from the first week of January 2021 to the last week of June 2021 across both environmental types. An asterisk * indicates larval pools were collected. Total number of ticks does not include larval pool count; Table S4: Published NCBI accession #s per each microorganism identified by PCR, n-PCR, or h-PCR. If the same microorganism was identified using different genes or in more than one tick species, separate NCBI accession #s were retrieved. The percent sequence identity to most closely related published species sequences are given only for reported genus level matches as species sequences matched 99% or higher to the species in GenBank. * Our ompA *Rickettsia* sp. sample from *I. affinis* was unable to be verified in NCBI blast, due to inclusion of a base pair shift, which inserts a stop codon in the translated protein sequence; Figure S3: Network graph indicating dual and triple infections between microorganisms in *Amblyomma americanum* adults and nymphs collected in greenspaces in Alachua County, Florida, USA. Color of line indicated number of ticks with co-infections, yellow *n* = 27, indigo *n* = 1; Table S5: Model results evaluating the influence of landscape variables on tick abundance. Best-fitting negative binomial GLMs (i.e., lowest AIC and all models with 2 ΔAIC value < 2) for (a) *Amblyomma americanum* and (b) *Ixodes scapularis* abundance. The ΔAIC for the null model is also indicated. *** indicates *p* < 0.01, ** indicates *p* < 0.05, and * indicates *p* < 0.1. The standard error for each estimate is indicated in parentheses. The development gradient represents the developed-to-natural landcover gradient using 500 m buffers for each site. Habitat type compares manicured sites to natural sites. Results are presented for models including all sites (full) and models excluding outlier sites (screened). The ‘screened’ dataset for *A. americanum* had two outlier sites removed, and the ‘screened’ dataset for *I. scapularis* has one outlier site removed; Table S6: Scale of effect on tick abundance. Generalized linear model results showing the response of abundance for (a) *Amblyomma americanum* and (b) *Ixodes scapularis* to the effects of the development gradient across all spatial scales; Table S7: Model results evaluating the influence of landscape variables on infected ticks and pathogen richness. Best-fitting negative binomial GLMs (i.e., lowest AIC and all models with 2 ΔAIC value < 2) for (a) the presence of infected tick, (b) the abundance of infected ticks, and (c) pathogen richness. The ΔAIC for the null model is also indicated. *** indicates *p* < 0.01, ** indicates *p* < 0.05, and * indicates *p* < 0.1. The standard error for each estimate is indicated in parentheses. The development gradient represents a developed-to-natural landcover gradient using 500 m buffers for each site. Habitat type compares manicured sites to natural site.
