# Supplementary material for: Ticks and Tick-Borne Pathogens in Recreational Greenspaces in North Central Florida, USA
Source: Microorganisms. 2023 Mar 15;11(3):756. doi: 10.3390/microorganisms11030756 (PMC10057063; doi:10.3390/microorganisms11030756)
Supplement: Supplementary file 1 [file microorganisms-11-00756-s001.zip › microorganisms-2272175-supplementary.pdf]

Supplementary Materials

Supplementary Table S1. Reclassification of Florida Land Cover Classification System. The Class ID and Class were obtained from the original Florida Land Cover Classification System (2018). Reclass and Reclass ID represent the land cover reclassification used for this study. In this reclassification, forest refers to any upland habitat that is not urbanized. In addition to canopied habitats, this classification can include sinkholes forested lots in otherwise urban landscapes, and pastures but agricultural land like row crops were separately classified.

Class ID	Class	Reclass	Reclass ID
1110	Upland Hardwood Forest	Forest	2
1120	Mesic Hammock	Forest	2
1123	Live Oak	Forest	2
1124	Pine - Mesic Oak	Forest	2
1150	Xeric Hammock	Forest	2
1210	Scrub	Forest	2
1214	Coastal Scrub	Forest	2
1220	Upland Mixed Woodland	Forest	2
1230	Upland Coniferous	Forest	2
1231	Upland Pine	Forest	2
1240	Sandhill	Forest	2
1311	Mesic Flatwoods	Forest	2
1312	Scrubby Flatwoods	Forest	2
1400	Mixed Hardwood-Coniferous	Forest	2
1410	Successional Hardwood Forest	Forest	2
1500	Shrub and Brushland	Forest	2
1710	Sinkhole	Forest	2
1730	Limestone Outcrop	Forest	2
1800	Cultural - Terrestrial	Lawn	3
1810	Mowed Grass	Lawn	3
1821	Low Intensity Urban	Residential	4
1822	High Intensity Urban	Residential	4
1831	Rural Open	Forest	2
1832	Rural Structures	Residential	4
1840	Transportation	Commercial	1
1841	Roads	Commercial	1
1850	Communication	Commercial	1
1860	Utilities	Commercial	1
1870	Extractive	Commercial	1
1872	Sand & Gravel Pits	Commercial	1

1873	Rock Quarries	Commercial	1
1877	Spoil Area	Commercial	1
1880	Bare Soil/Clear Cut	Commercial	1
2111	Wet Prairie	Wetland	6
2112	Mixed Scrub-Shrub Wetland	Wetland	6
2120	Marshes	Wetland	6
2121	Isolated Freshwater Marsh	Wetland	6
2140	Floating/Emergent Aquatic Vegetation	Wetland	6
2211	Cypress	Wetland	6
2215	Floodplain Swamp	Wetland	6
2220	Other Coniferous Wetlands	Wetland	6
2221	Wet Flatwoods	Wetland	6
2231	Baygall	Wetland	6
2232	Hydric Hammock	Wetland	6
2233	Mixed Wetland Hardwoods	Wetland	6
2240	Mixed Hardwood Coniferous Swamps	Wetland	6
2440	Clearcut Wetland	Wetland	6
3000	Lacustrine	Water	5
3100	Natural Lakes and Ponds	Water	5
3111	Clastic Upland Lake	Water	5
3113	Flatwoods/Prairie/Marsh Lake	Water	5
3114	River Floodplain Lake/Swamp Lake	Water	5
3115	Sinkhole Lake	Water	5
3117	Sandhill Lake	Water	5
3200	Cultural - Lacustrine	Water	5
3210	Artificial/Farm Pond	Water	5
3211	Aquacultural Ponds	Water	5
3220	Artificial Impoundment/Reservoir	Water	5
3230	Quarry Pond	Water	5
3240	Sewage Treatment Pond	Water	5
3250	Stormwater Treatment Areas	Water	5
3260	Industrial Cooling Pond	Water	5
4000	Riverine	Water	5
4100	Natural Rivers and Streams	Water	5
4120	Blackwater Stream	Water	5
4130	Spring-run Stream	Water	5
4140	Seepage Stream	Water	5
4220	Ditch/Artificial Intermittent Stream	Water	5
18211	Urban Open Land	Forest	2

18212	Residential, Low Density	Residential	4
18213	Grass	Lawn	3
18221	Residential, Med. Density - 2-5 Dwelling Units/AC	Residential	4
18222	Residential, High Density > 5 Dwelling Units/AC	Residential	4
18223	Commercial and Services	Commercial	1
18224	Industrial	Commercial	1
18225	Institutional	Commercial	1
18311	Rural Open Forested	Forest	2
18312	Rural Open Pine	Forest	2
18332	Orchards/Groves	Agriculture	0
18334	Vineyard and Nurseries	Agriculture	0
21211	Depression Marsh	Wetland	6
21212	Basin Marsh	Wetland	6
22131	Dome Swamp	Wetland	6
22132	Basin Swamp	Wetland	6
22211	Hydric Pine Flatwoods	Wetland	6
22311	Bay Swamp	Wetland	6
22323	Cabbage Palm Hammock	Wetland	6
22331	Bottomland Forest	Wetland	6
182111	Urban Open Forested	Forest	2
182112	Urban Open Pine	Forest	2
182131	Parks and Zoos	Lawn	3
182132	Golf courses	Lawn	3
182133	Ballfields	Lawn	3
182134	Cemeteries	Lawn	3
182135	Community rec. facilities	Lawn	3
183311	Row Crops	Agriculture	0
183312	Field Crops	Agriculture	0
183313	Improved Pasture	Agriculture	0
183314	Unimproved/Woodland Pasture	Forest	2
183321	Citrus	Agriculture	0
183323	Pecan	Agriculture	0
183332	Coniferous Plantations	Forest	2
183341	Tree Nurseries	Agriculture	0
183343	Ornamentals	Agriculture	0
183351	Feeding Operations	Agriculture	0
183352	Specialty Farms	Agriculture	0
221312	Gum Pond	Wetland	6
1833111	Irrigated Cropland	Agriculture	0

1833151	Fallow Cropland	Agriculture	0
1833321	Wet Coniferous Plantation	Forest	2

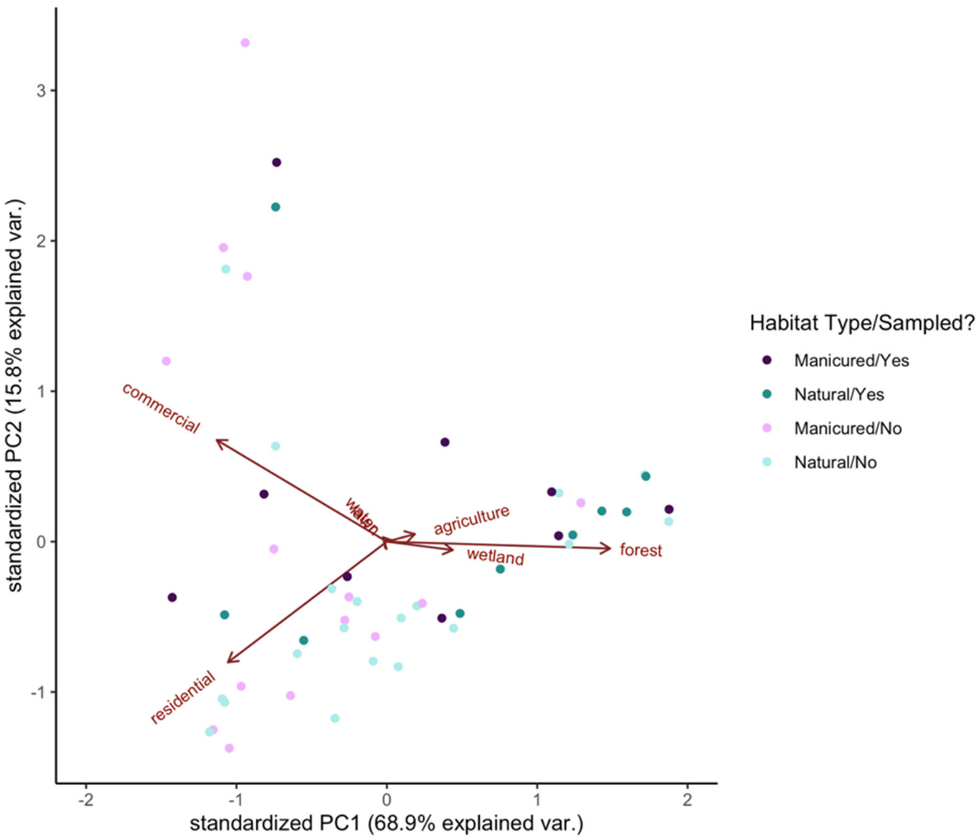

Supplementary Figure S1. PCA biplot of all sampling sites considered in this study. PC1, which represents nearly 70% of variation in landcover composition surrounding sites using a 1km buffer, represents a developed (negative PC1 values) to undeveloped (positive values of PC1) gradient. Sites sampled in this study are represented in dark purple and dark green.

Supplemental Table S2. List of recreational greenspaces sampled in Alachua County, Florida with sampling site habitat type designation (natural or manicured), number of ticks collected, species of ticks identified, and microorganisms present at each site.

Greenspace	Internal Sampling site habitat designation	Managed by	N number of ticks (adults, nymphs) collected	Species of tick collected	Larvae Collected (Yes/No) (n = # of Pools)	Microorganisms present by genus or species if of medical/veterinary importance
Harmonic Woods	Natural	The University of Florida	2	*A. americanum,*<br><br>*I. scapularis*	No	*None*<br><br>*None*
29th Road Nature Park	Natural	City of Gainesville Parks, Recreation, & Cultural Affairs	7	*A. americanum,*<br><br>*H. leporispalustris*	No	*Rickettsia amblyommatis*<br><br>*None*
John Mahon Nature Park	Natural	City of Gainesville Parks, Recreation, & Cultural Affairs	4	*A. americanum,*<br><br>*I. scapularis*	No	*Rickettsia amblyommatis*<br>*Ehrlichia Panola Mountain*<br><br>*Rickettsia* sp. *endosymbiont I. scap*
Sweetwater Preserve	Natural	Alachua County Environmental Protection Department, Office of Conservation Lands, Alachua County Forever	75	*A. americanum,*<br><br>*I. scapularis*	No	*Rickettsia amblyommatis*<br>*Babesia* sp.<br>*Babesia* sp. *(Coco)*<br>*Ehrlichia* sp. *Panola Mountain*<br><br>*Babesia odocoilei*<br>*Rickettsia* sp. *endosymbiont I. scap*
Split Rock Conservation Area	Natural	City of Gainesville Parks, Recreation, & Cultural Affairs	440	*A. americanum,*<br><br>*D. variabilis,*<br><br>*I. scapularis*	Yes (n = 5)	*Rickettsia amblyommatis*<br>*Hepatozoon* sp. *A*<br>*Babesia* sp. (Coco)<br>*Theileria cervi*<br>*Ehrlichia chaffeensis*<br>*Ehrlichia* sp. *Panola Mountain*<br>*Ehrlichia ewingii*<br>*Rickettsia parkeri*<br>*Borrelia lonestari*<br>*Hepatozoon* sp. *A*<br>*Babesia* sp.<br><br>*Rickettsia rhiphicephali*<br><br>*Rickettsia* sp. *endosymbiont I. scap*<br>*Cryptoplasma* sp.

Morningside Nature Center A	Natural	City of Gainesville Parks, Recreation, & Cultural Affairs	588	*A. americanum,*<br><br><br><br><br><br><br>*D. variabilis,*<br><br><br>*I. scapularis*	Yes (n = 3)	*Rickettsia amblyommatis*<br>*Theileria cervi*<br>*Ehrlichia* sp. *Panola Mountain*<br>*Ehrlichia ewingii*<br>*Ehrlichia chaffeensis*<br>*Babesia* sp.<br>*Hepatozoon* sp. *A*<br><br>*Rickettsia rhiphicephali*<br>*Theileria cervi*<br><br>*Babesia odocoilei*<br>*Rickettsia sp. endosymbiont I. scap*<br>*Cryptoplasma (Allocryptoplasma)* sp.
San Felasco Hammock Preserve State Park	Natural	Florida Department of Environmental Protection, Division of Recreation and Parks, Florida Park Service	147	*A. americanum,*<br><br><br><br><br><br>*D. variabilis,*<br><br>*I. scapularis*	Yes (n = 1)	*Rickettsia amblyommatis*<br>*Theileria cervi*<br>*Ehrlichia chaffeensis*<br>*Ehrlichia* sp. *Panola Mountain*<br>*Hepatozoon* sp. *A*<br><br>*None*<br><br>*None*
Paynes Prairie Preserve State Park	Natural	Florida Department of Environmental Protection, Division of Recreation and Parks, Florida Park Service	302	*A. americanum,*<br><br><br><br><br><br><br><br><br><br><br>*D. variabilis,*<br><br>*I. scapularis*	Yes (n = 3)	*Rickettsia amblyommatis*<br>*Hepatozoon* sp. *A*<br>*Theileria cervi*<br>*Ehrlichia chaffeensis*<br>*Borrelia lonestari*<br>*Theileria cervi*<br>*Ehrlichia* sp. *Panola Mountain*<br>*Ehrlichia ewingii*<br>*Babesia* sp. *(Coco)*<br>*Babesia* sp.<br><br>*None*<br><br>*Rickettsia* sp. *endosymbiont I. scap*
Hatchet Creek Wildlife Management Area	Natural	St. Johns River Water Management District	102	*A. americanum,*<br><br>*A. maculatum,*<br><br>*I. affinis,*<br><br>*I. scapularis*	Yes (n = 6)	*Rickettsia amblyommatis*<br>*Theileria cervi*<br><br>*Rickettsia andeanae*<br><br>*Rickettsia* sp. *I. aff.*<br><br>*Rickettsia sp. endosymbiont I. scap*<br>*Babesia odocoieli*<br>*Cryptoplasma (Allocryptoplasma)* sp.<br>*Hepatozoon* sp. *B*
Reserve Park	Manicured	City of Gainesville	2	*A. americanum*	No	*Theileria cervi*

		Parks, Recreation, & Cultural Affairs				
University Gardens	Manicured	The University of Florida	2	*I. scapularis*	No	*None*
Hogtown Creek Headwaters Nature Park	Manicured	City of Gainesville Parks, Recreation, & Cultural Affairs	42	*A. americanum,*<br><br>*A. maculatum*	Yes (n = 1)	*Rickettsia amblyommatis*<br>*Ehrlichia chaffeensis*<br>*Borrelia lonestari*<br>*Babesia* sp.<br>*Theileria cervi*<br><br>*None*
Possum Creek Park	Manicured	City of Gainesville Parks, Recreation, & Cultural Affairs	5	*A. americanum*	No	*Rickettsia amblyommatis*
Fred Cone Park	Manicured	City of Gainesville Parks, Recreation, & Cultural Affairs	15	*A. americanum,*<br><br>*D. variabilis*		*Rickettsia amblyommatis*<br>*Babesia* sp.<br>*Babesia* sp. (Coco)<br><br>*None*
Green Acres Park	Manicured	City of Gainesville Parks, Recreation, & Cultural Affairs	0	*NA*	No	*NA*
Morningside Nature Center B	Manicured	City of Gainesville Parks, Recreation, & Cultural Affairs	45	*A. americanum,*<br><br>*I. scapularis*	No	*Rickettsia amblyommatis*<br>*Borrelia lonestari*<br>*Babesia* sp.<br>*Theileria cervi*<br><br>*Rickettsia sp. endosymbiont I. scap*<br>*Cryptoplasma (Allocryptoplasma) sp.*
Dudley Farm Historic State Park	Manicured	Florida Department of Environmental Protection, Division of Recreation and Parks, Florida Park Service	31	*A. americanum,*<br><br>*I. scapularis,*<br><br>*D. variabilis*	No	*Rickettsia amblyommatis*<br>*Babesia* sp.<br>*Hepatozoon* sp. *A*<br>*Borrelia lonestari*<br>*Babesia* sp. (Coco)<br><br>*Babesia odocoilei*<br><br>*None*
Longleaf Flatwoods Reserve	Manicured	St. Johns River Water	17	*A. americanum*	Yes (n = 2)	*Rickettsia amblyommatis*<br>*Rickettsia parkeri*<br>*Borrelia lonestari*

		Managemen t District				*Theileria cervi*

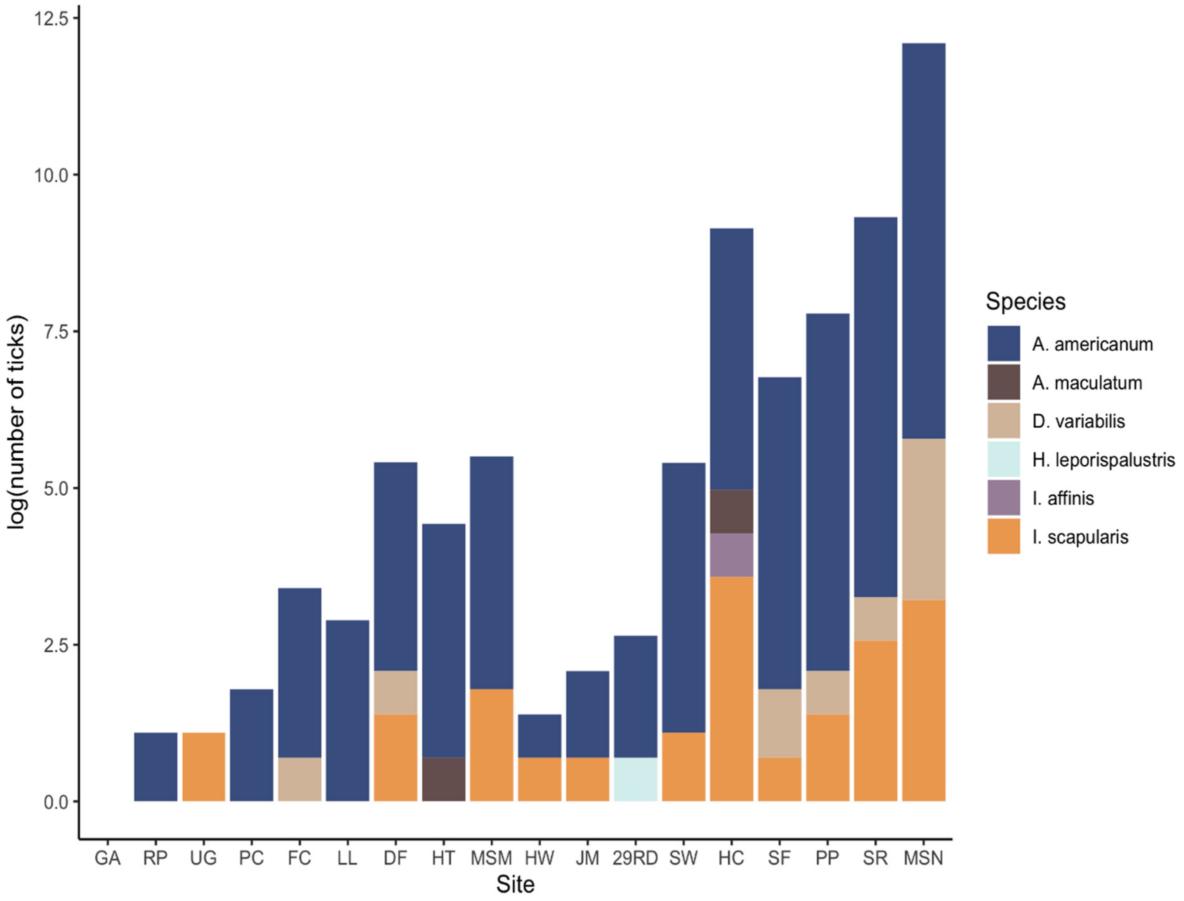

Supplementary Figure S2. Total tick abundance (log transformed) by species collected in across all 18 sampling sites. First 9 sites are designated as manicured, while the last 9 sites are designated as natural sites.

Supplementary Table S3. Total number of tick species collected per life stage from the first week of January 2021 to the last week of June 2021 across both environmental types. An asterisk * indicates larval pools were collected. Total number of ticks does not include larval pool count.

Tick species and life stage	Natural	Manicured	Total
A. americanum adult	629	44	**673**
A. americanum nymph	940	102	**1042**
A. americanum larvae	*(18 pools)	*(3 pools)	***(21 pools)**
A. maculatum nymph	1	1	**2**
D. variabilis adult	16	2	**18**
H. leporispalustris nymph	1	0	**1**
I. affinis adult	1	0	**1**
I. scapularis adult	79	10	**89**
Total	**1667**	**159**	**1826**

Supplementary Table S4. Published NCBI accession #s per each microorganism identified by PCR, n-PCR, or h-PCR. If the same microorganism was identified using different genes or in more than one tick species, separate NCBI accession #s were retrieved. The percent sequence identity to most closely related published species sequences are given only for reported genus level matches as species sequences matched 99% or higher to species in GenBank. *Our ompA *Rickettsia* sp. sample from *I. affinis* was unable to be verified in NCBI blast due to inclusion of base pair shift which inserts stop codon in translated protein sequence.

Tick species	Microorganism	Gene [NCBI Accession #]	% Identity to species in NCBI for genus matches.
A. americanum	*Babesia* sp.	*18s [OM865359]*	*100% Babesia sp. NYT-435 [MW665118] & Babesia sp. H10 clone 3 [HQ264119]*
	Babesia sp. (Coco)	*18s [OM865360]*	---
	Borrelia lonestari	*flaB [OM960690]*	---
	Cytauxzoon felis	*18s [OM865361]*	---
	Ehrlichia ewingii	*groEL [OM960692]*	---
	Ehrlichia chaffeensis	*groEL [OM960693]*	---
	Panola Mtn. Ehrlichia sp.	*groEL [OM960694]; gltA [OM960695]*	---
	Hepatozoon sp. A	*18s [OM865362]*	*96.7% Hepatoozon procyonis [MF685396]*
	Rickettsia amblyommatis	*ompA [OM960698]*	---
	Rickettsia parkeri	*PLA [OM960702]*	---
	Theileria cervi	*18s [OM865364]*	---
A. maculatum	*Rickettsia andeanae*	*ompA [OM960699]; gltA [OM960696]*	---
D. variabilis	*Rickettsia rhiphicephali*	*ompA [OM960701]; gltA [OM960697]; PLA [OM960703]*	---
	Theileria cervi	*18s [OM865365]*	---
I. affinis	*Rickettsia* sp.	*ompA [*]; gltA [ON081007]*	*99% Rickettsia sp. clone BelizeIaff [KU001175] ompA & 99.2% uncultured Rickettsia sp. clone NP9 gltA [MT441702]*
I. scapularis	*Babesia odocoieli*	*18s [OM865358]*	---
	Cryptoplasma (Allocryptoplasma) sp.	*groEL [OM960691]; 16s [OM884475]*	*97.1% Candidatus Cryptoplasma californiense [KP276601] ompA & 100% Candidatus Cryptoplasma californiense [KP276585] 16s.*

	Hepatozoon sp. B	*18s [OM865363]*	*96.9% Hepatozoon* sp. *[MZ351069]*
	Rickettsia sp. *endosymbiont*	*ompA [OM960700]*	---

Amblyomma americanum (adults and nymphs)

Babesia sp.	*Babesia* sp. Coco	*Borrelia lonestari*	*Ehrlichia* sp. PanolaMtn	*Ehrlichia chaffeensis*	*Ehrlichia ewingii*	*Hepatozoon* sp.	*Rickettsia amblyommatis*	*Rickettsia parkeri*	*Theileria cervi*	
-	0	0	0	0	0	0	8	0	0	*Babesia* sp.
	-	0	0	0	0	0	7	0	0	*Babesia* sp. Coco
		-	0	0	0	0	4	0	0	*Borrelia lonestari*
			-	0	1	0	2	0	0	*Ehrlichia* sp. PanolaMtn
				-	0	0	2	0	1	*Ehrlichia chaffeensis*
					-	0	5	0	0	*Ehrlichia ewingii*
						-	12	0	0	*Hepatozoon* sp.
							-	2	27	*Rickettsia amblyommatis*
								-	0	*Rickettsia parkeri*
									-	*Theileria cervi*

Ixodes scapularis (adults)

Babesia odocoieli	*Cryptoplasma* sp.	*Hepatozoon* sp.	*Rickettsia* sp.	
-	1	0	2	*Babesia odocoieli*
	-	0	2	*Cryptoplasma* sp.
		-	1	*Hepatozoon* sp.
			-	*Rickettsia* sp.

Supplementary Figure S3. Matrices of bacterial co-infections in individual *Amblyomma americanum* and *Ixodes scapularis* ticks collected in Alachua County, Florida, USA from January to June 2021.

Supplementary Table S5. Model results evaluating the influence of landscape variables on tick abundance. Best-fitting negative binomial GLMs (i.e., lowest AIC and all models with 2 ΔAIC value < 2) for (a) *A. americanum* and (b) *I. scapularis* abundance. The ΔAIC for the null model is also indicated. *** indicates p <0.01, ** indicates p <0.05, and * indicates p<0.1. The standard error for each estimate is indicated in parentheses. The development gradient represents the developed to natural landcover gradient using 500m buffers for each site. Habitat type compares manicured sites to natural sites. Results are presented for models including all sites (full) and models excluding outlier sites (screened). The 'screened' dataset for *A. americanum* had two outlier sites removed, and the 'screened' dataset for *I. scapularis* has one outlier site removed.

Species	Dataset	Intercept	Habitat Type	Development Gradient	AIC	delta AIC	Pseudo R^2
(a) *A. americanum*							
	full	2.461*** (0.377)	1.238*** (0.525)	0.945*** (0.165)	170.4	0	62.1
		3.144*** (0.305)		1.129*** (0.182)	172.4	2	53.3
		4.556*** (0.405)			186.4	16	0
	screened	1.868*** (0.315)	1.240*** (0.413)	1.003*** (0.131	134.6	0	80.4
		4.355*** (0.439)			158.4	23.8	0
(b) *I. scapularis*							
	full	-0.169 (0.531)	1.371** (0.666)	0.620*** (0.216)	83.6	0	49.0
		0.645 (0.410)		0.752*** (0.242)	84.8	1.2	37.1
		1.598*** (0.438)			90.7	7.1	0
	screened	-0.765 (0.628)	1.721** (0.716)	0.825*** (0.248)	76.0	0	58.5
		1.633*** (0.469)			85.5	9.5	0

Supplementary Table S6. "Scale of effect" on tick abundance. Generalized linear model results showing the response of abundance for (a) *Amblyomma americanum* and (b) *Ixodes scapularis* to the effects of the development gradient across all spatial scales.

Model (Spatial Scale)	Intercept	Estimate	logLik	delta AICc
(a) *Amblyomma americanum*				
PC1 (500m)	3.525	0.829	-1017	0
PC1 (250m)	3.884	-0.844	-1099	164
PC1 (1km)	4.062	0.429	-1374	714
PC1 (2km)	4.244	0.306	-1489	945
PC1 (3km)	4.416	0.175	-1683	1333
PC1 (4km)	4.474	0.119	-1723	1436
(b) *Ixodes scapularis*				
PC1 (500m)	0.487	0.874	-80	0
PC1 (1km)	0.773	0.603	-87	12
PC1 (2km)	0.963	0.477	-89	17
PC1 (250m)	1.019	-0.770	-91	21
PC1 (4km)	1.246	0.305	-103	44
PC1 (3km)	1.224	0.331	-103	46

Supplementary Table S7. Model results evaluating the influence of landscape variables on infected ticks and pathogen richness. Best-fitting negative binomial GLMs (i.e., lowest AIC and all models with 2 ΔAIC value < 2) for (a) the presence of infected tick, (b) the abundance of infected ticks, and (c) pathogen richness. The ΔAIC for the null model is also indicated. *** indicates p <0.01, ** indicates p <0.05, and * indicates p<0.1. The standard error for each estimate is indicated in parentheses. The development gradient represents a developed to natural landcover gradient using 500m buffers for each site. Habitat type compares manicured sites to natural sites.

Response Variable	Intercept	Habitat Type	Development Gradient	AIC	delta AIC	Pseudo R^2
(a) presence of infected tick						
	-1.591 (1.198)	3.835 (2.702)	1.690* (0.971)	17.1	0	54.9
	0.223 (0.474)			26.7	7.2	0
(b) abundance of infected ticks						
	-1.187* (0.625)	1.945*** (0.688)	0.608*** (0.211)	64.5	0	61.0
	0.916** (0.401)			75.5	11	0
(c) pathogen richness						
	-1.082** (0.550)	1.238** (0.600)	0.493** (0.197)	52.0	0	54.4
	0.288 (0.327)			61.3	9.3	0
